# Changing trends of disease burden of stroke from 1990 to 2019 and its predictions among the Chinese population

**DOI:** 10.3389/fneur.2023.1255524

**Published:** 2023-10-04

**Authors:** Dong Liang, Qing Guan, Minqing Huang, Yiyu He, Yangjiang Ou, Min Chen, Xiaoxin Zheng, Xiuquan Lin

**Affiliations:** ^1^The School of Health Management, Fujian Medical University, Fuzhou, Fujian, China; ^2^The School of Public Health, Fujian Medical University, Fuzhou, Fujian, China; ^3^Department of Cardiology, Renmin Hospital of Wuhan University, Wuhan, Hubei, China; ^4^Cardiovascular Research Institute, Wuhan University, Wuhan, Hubei, China; ^5^Hubei Key Laboratory of Cardiology, Wuhan, Hubei, China; ^6^“The 14th Five-Year Plan” Application Characteristic Discipline of Hunan Province (Clinical Medicine), Hunan Provincial Key Laboratory of the Traditional Chinese Medicine Agricultural Biogenomics, Changsha Medical University, Changsha, Hunan, China; ^7^Department for Chronic and Noncommunicable Disease Control and Prevention, Fujian Provincial Center for Disease Control and Prevention, Fuzhou, Fujian, China

**Keywords:** stroke, disease burden, temporal trend, risk factor, prediction

## Abstract

**Objective:**

This study aimed to understand the temporal trends in the disease burden of stroke and its attributable risk factors in China, along with the future trends in the next 25 years, that is important for effective prevention strategies and improvement, and to provide new insights into the age- and sex-specific incidence, prevalence, mortality, disability-adjusted life-years (DALYs) and their trends from 1990 to 2019, and the prediction in the next 25 years.

**Methods:**

The Global Burden of Disease Study (2019) was used to extract the data on age- and sex-specific incidence, mortality, and disability-adjusted life-years (DALYs) of stroke in China, 1990–2019. We estimated the estimated annual percentage change (EAPC) to access the temporal trends of the disease burden of stroke. The R package called Nordpred was used to perform an age-period-cohort analysis to predict the prevalence of stroke.

**Results:**

The number of incidence cases, deaths, and DALYs of stroke increased from 1990 to 2019. Overall downward trends were observed in the age-standardized incidence rate (ASIR) from 1990 to 2019. Significant temporal trends in mortality and DALYs of stroke were observed. High systolic blood pressure, smoking, and high-sodium diet were the main driving forces for stroke. The DALYs lost attributable to smoking were different for male and female patients. In the next 25 years, the number of new cases and deaths from stroke should continue to increase. The ASIR and age-standardized mortality rate (ASMR) should show a downward trend among male and female patients.

**Conclusion:**

Despite the overall rates of stroke declined over the period from 1990 to 2019, the absolute number of people affected by stroke has substantially increased. There has been a substantial increase in the burden of stroke due to risk factors and will continue to increase in the next 25 years.

## Introduction

Stroke is a global health issue, which can either be hemorrhagic (a rupture of a blood vessel) or ischemic (an occlusion of a blood vessel). Annually, 15 million people suffer a stroke worldwide, of which 5 million die, making it the second leading cause of death globally (1) and another 5 million are left permanently disabled. Stroke becomes the leading cause of longtime disability, especially in low-income and middle-income countries (2). The Global Burden of Disease Study (GBD) 2019 estimated that deaths caused by stroke in China reached ~4 million in 2019, and the incidence of stroke, including hemorrhagic stroke (IS) and ischemic stroke (HS), showed a general increasing trend in the past years (3). The number of stroke patients in China is likely to rise as a result of lifestyle and demographic changes, as well as inadequate control of major risk factors for stroke (4). Despite its implications and comorbidities, stroke still receives relatively less research or public attention. Stroke prevention and treatment are urgently needed in China due to its high incidence.

Both environmental and genetic factors contribute to ischemic and hemorrhage stroke disease. Some of these factors are not modifiable, such as age, gender, and family history, while others are potentially modifiable, such as hypertension, smoking, and higher sodium intake. It is possible to control and prevent strokes by modifying these potentially modifiable factors. There is a continuous, consistent, and independent relationship between blood pressure and the risk of developing stroke. Observational studies indicate that the risk of death from both ischemic heart disease and stroke increases beginning at systolic blood pressure as low as 115 mmHg. The mortality from stroke doubles with each increment of 20 mmHg systolic blood pressure (5). In a plethora of studies over the years, smoking has been identified as an independent risk factor for stroke. The relative risk for stroke ascribed to cigarette smoking is 1.5 (6). Dietary salt increases the risk of death from stroke, especially in overweight individuals, and higher sodium intake is associated with ~89% increased risk for stroke mortality (7).

The latest Global Burden of Diseases (GBD) study (2019) has provided new epidemiological data on the incidence, mortality, and disability-adjusted life-years (DALYs) of stroke from 1990 to 2019, enabling us to provide updated estimates of the prevalence and risk factors for stroke in China. However, to the best of our knowledge, no published article has yet described the disease burden and attributable risk factors of stroke by age, sex, and year, and the future trends in the next 25 years of the disease burden of stroke in China. Therefore, we conducted a comprehensive and rigorously designed assessment of stroke incidence, mortality, and DALYs, stratified by age and gender. We also analyzed primary risk factors for stroke and forecasted its incidence and mortality rates in China over the next 25 years. The analysis of epidemiological trends and major risk factors of stroke as well as the prediction of future epidemiological trends in our study will be of great significance in reducing the incidence and mortality of stroke.

## Materials and methods

The data of age- and sex-specific incidence, mortality, and disability-adjusted life-years (DALYs) of ischemic and hemorrhage stroke in China, 1990–2019, were derived from the Global Burden of Disease Study (2019). The estimated annual percentage change (EAPC) was estimated to access the temporal trends of disease burden of ischemic and hemorrhage stroke, and the R package called Nordpred was used to perform an age-period-cohort analysis to predict the numbers and rates of incidence and mortality for ischemic and hemorrhage stroke in the next 25 years.

### Data sources

The data on incidence, mortality, and DALYs of ischemic and hemorrhage stroke were downloaded from the website of Institute for Health Metrics and Evaluation (IHME) (http://ghdx.healthdata.org/gbd-results-tool), and the rules of this selecting data were as follows: location name was “China,” the cause was “stroke,” and measures were “incidence,” “mortality,” and “DALYs.” These indicators were calculated with 95% uncertainty intervals (95% UIs).

The WHO World Standard Population Distribution (2000–2025) was used as the standard population. The United Nations World Population Prospects 2019 Revision (https://population.un.Org/WPP/Download/Standard/Population/) was used as the prediction population. Ethics approval was not required as this study was based on publicly available data (GBD, 2019), and no personal data were collected.

### Evaluation of ischemic and hemorrhage stroke burden

Estimates of the incidence and prevalence of ischemic and hemorrhage stroke were calculated with the DisMod-MR2.1 (disease-model-Bayesian meta-regression) modeling tool. DisMOd-MR is a Bayesian geospatial disease modeling software that uses various disease parameters, the epidemiological relationships between these parameters, and geospatial relationships to estimate incidence and prevalence. All available high-quality data on incidence, prevalence, and mortality were used to estimate the non-fatal ischemic and hemorrhage stroke burden. All-cause and cause-specific mortality for ischemic and hemorrhage stroke were estimated using the Cause of Death Ensemble modeling. DALYs were the sum of years lived with disabilities (YLDs) and years of life lost (YLLs). YLDs were calculated by multiplying the prevalence with the corresponding disability weights. YLLs were calculated by multiplying observed deaths for a specific age by global age-specific reference life expectancy. In the study, 95% UIs capturing both random and systematic error in statistical modeling were calculated for all estimates. For the risk factors, the comparative risk assessment (CRA) framework was used to estimate the proportion of DALYs attributable to three well-established risk factors for ischemic and hemorrhage stroke by age and sex: high fasting glucose, high systolic blood pressure, and smoking. The detailed study methods of GBD 2019 have been reported in previous studies (8–10).

### Statistical analysis

The incidence, mortality, and DALYs of ischemic and hemorrhage stroke were performed by age group, sex, and year. The temporal trends for these indicators from 1990 to 2019 were plotted. The age was divided into 18 age groups at 5 years, and 0–39 years were combined into one age group. The time trends of age-standardized incidence (ASIR), age-standardized mortality (ASMR), and age-standardized DALY rates were described by the estimated annual percentage change (EAPC) which was calculated from a regression model with the natural logarithm of the rate, that is, ln(rate) = α + β × (calendar year) + ε. EAPC was defined as 100 × (exp (β) −1). Its 95% confidence interval (95% CI) was also generated from the fitted model.

The power5 APC model of the R package called Nordpred which has been shown to perform well in predicting the trend of disease incidence and mortality (11, 12) was used to predict the number and rate of ischemic and hemorrhage stroke incidence and mortality in the next 25 years. Moreover, we estimated the number and rates of ischemic and hemorrhage stroke events by assuming that the events for ischemic and hemorrhage stroke remained stable, decreased, and increased by 1% per year based on the observed data of ischemic and hemorrhage stroke in 2019 in order to facilitate comparison with predicted results. We used the ggplot2 packages from the R program (Version 4.1.2; R core team, R Foundation for Statistical Computing, Vienna, Austria) to perform the visualization of the results.

## Results

### Incidence, mortality, and DALYs of ischemic and hemorrhage stroke in 2019

In 2019, the number of incidence cases and ASIR of ischemic and hemorrhage stroke among the total Chinese population were 3935.18 thousand (95% UI: 3431.72, 4579.87) and 200.84 per 100,000 (95% UI: 176.95, 230.84), respectively (Table 1). Ischemic and hemorrhage stroke contributed to 2189.18 thousand (95% UI: 1885.90, 2513.77) deaths in 2019, and ASMR was 127.25 per 100,000 (95% UI: 110.21, 144.89) among the total Chinese population (Table 2). Ischemic and hemorrhage stroke caused 45949.13 thousand (95% UI: 39813.51, 52335.53) DALYs in 2019, and the age-standardized rate of DALYs was 2412.52 per 100,000 (95% UI: 2102.92, 2742.48) (Table 3). The number and age-standardized rates of incidence, mortality, and DALYs are about the same for male and female patients (Tables 1–3).

**Table 1:** Number of incidence cases and incidence rate of ischemic and hemorrhage stroke in China in 1990 and 2019 and EAPC from 1990 to 2019.

**Characteristics**	**1990**		**2019**		**1990–2019**
	**Incidence cases [**×**10**^3^ **(95% UI)]**	**Incidence rate [per 1, 00, 000 (95% UI)]**	**Incidence cases [**×**10**^3^ **(95% UI)]**	**Incidence rate [per 1, 00, 000 (95% UI)]**	**EAPC in incidence rate [% (95% CI)]**
Overall^a^	1760.44 (1560.97, 2005.21)	221.51 (196.81, 249.61)	3935.18 (3431.72, 4579.87)	200.84 (176.95, 230.84)	−1.31 (−3.41, 0.85)
**Sex** ^a^					
Male	868.03 (769.11, 993.05)	227.92 (202.83, 257.34)	1950.98 (1713.53, 2260.51)	209.75 (185.84, 239.39)	−1.20 (−3.35, 1.01)
Female	892.41 (791.42, 1019.26)	216.45 (192.14, 245.19)	1984.2 (1719.09, 2322.54)	194.53 (169.65, 225.19)	−1.43 (−3.51, 0.69)
**Age at diagnosis**^b^ **(year)**					
0–14	52.23 (33.77, 76.41)	16.17 (10.46, 23.66)	31.02 (19.76, 46.63)	13.80 (8.79, 20.74)	−0.35 (−2.42, 1.76)
15–19	17.88 (10.91, 27.05)	14.09 (8.60, 21.32)	9.29 (5.41, 14.68)	12.36 (7.20, 19.54)	−0.81 (−2.21, 0.61)
20–24	23.21 (16.22, 32.60)	17.53 (12.25, 24.62)	12.32 (8.22, 18.28)	15.05 (10.04, 22.33)	−1.15 (−2.47, 0.20)
25–29	27.28 (18.89, 38.75)	24.75 (17.14, 35.17)	22.82 (14.78, 34.81)	20.61 (13.35, 31.44)	−1.37 (−2.73, 0.00)
30–34	33.15 (25.16, 43.19)	37.45 (28.42, 48.79)	38.97 (28.41, 53.07)	30.19 (22.01, 41.11)	−1.51 (−2.90, −0.10)
35–39	50.16 (36.26, 68.44)	54.81 (39.62, 74.79)	43.79 (32.31, 58.53)	43.41 (32.02, 58.01)	−1.54 (−2.98, −0.08)
40–44	67.15 (53.49, 83.15)	99.86 (79.55, 123.65)	79.26 (63.82, 98.76)	77.97 (62.79, 97.16)	−1.55 (−3.14, 0.05)
45–49	89.45 (66.09, 115.75)	172.94 (127.78, 223.80)	162.49 (121.27, 213.82)	133.88 (99.92, 176.18)	−1.55 (−3.25, 0.19)
50–54	135.62 (108.49, 164.95)	283.72 (226.96, 345.07)	304.26 (247.94, 369.49)	243.20 (198.19, 295.34)	−1.25 (−3.14, 0.68)
55–59	186.22 (140.48, 244.07)	428.47 (323.23, 561.57)	386.79 (286.65, 508.44)	407.83 (302.25, 536.1)	−0.96 (−3.04, 1.16)
60–64	223.72 (178.07, 278.53)	631.62 (502.72, 786.36)	505.13 (398.15, 635.25)	643.03 (506.84, 808.66)	−0.91 (−3.15, 1.38)
65–69	244.78 (177.31, 325.91)	894.41 (647.9, 1190.87)	663.85 (468.8, 903.16)	943.19 (666.07, 1283.19)	−0.98 (−3.35, 1.44)
70–74	231.55 (183.45, 293.70)	1227.95 (972.86, 1557.56)	612.33 (477.76, 794.97)	1279.53 (998.33, 1661.17)	−1.05 (−3.41, 1.37)
75–79	186.56 (144.17, 235.81)	1635.02 (1263.5, 2066.59)	498.40 (389.29, 627.74)	1669.87 (1304.31, 2103.22)	−1.11 (−3.45, 1.28)
80–84	122.25 (97.6, 150.43)	2168.07 (1730.89, 2667.74)	357.78 (282.01, 449.01)	1876.40 (1479.02, 2354.85)	−1.59 (−3.88, 0.76)
85–89	53.92 (43.03, 68.19)	2812.1 (2243.81, 3556.03)	159.43 (130.38, 192.61)	1874.60 (1533.11, 2264.80)	−2.30 (−4.53, −0.02)
90–94	12.78 (9.49, 17.23)	3428.42 (2545.6, 4623.93)	39.80 (30.54, 49.49)	1773.69 (1360.92, 2205.35)	−3.06 (−5.24, −0.82)
95+	2.53 (1.70, 3.59)	4068.08 (2735.78, 5762.46)	7.46 (4.97, 10.14)	1671.05 (1111.93, 2270.55)	−3.61 (−5.77, −1.40)

EAPC, estimated annual percentage change; 95% UI, 95% uncertainty interval; 95% CI, 95% confidence interval;

a age-standardized incidence rate;

b crude incidence rate in each age group.

**Table 2:** Number of deaths and mortality rate of ischemic and hemorrhage stroke in China in 1990 and 2019 and EAPC from 1990 to 2019.

**Characteristics**	**1990**		**2019**		**1990–2019**
	**Deaths cases [**×**10**^3^ **(95% UI)]**	**Mortality rate [per 100, 000 (95% UI)]**	**Deaths cases [**×**10**^3^ **(95% UI)]**	**Mortality rate [per 1, 00, 000 (95% UI)]**	**EAPC in mortality rate [% (95% CI)]**
Overall^a^	1377.09 (1220.67, 1564.24)	211.44 (187.68, 243.80)	2189.18 (1885.90, 2513.77)	127.25 (110.21, 144.89)	−2.85 (−4.68, −0.98)
**Sex** ^a^					
Male	712.83 (599.05, 837.93)	246.30 (212.62, 286.86)	1261.12 (1035.32, 1509.29)	170.32 (141.87, 200.18)	−2.26 (−4.14, −0.34)
Female	664.26 (567.00, 792.33)	188.28 (161.70, 224.20)	928.06 (747.28, 1117.12)	97.44 (78.87, 117.01)	−3.40 (−5.18, −1.59)
**Age at diagnosis** ^b^ **(year)**					
0–14	10.93 (7.46, 13.40)	3.39 (2.31, 4.15)	0.69 (0.55, 0.96)	0.31 (0.24, 0.43)	−7.43 (−9.24, −5.59)
15–19	3.32 (2.74, 3.96)	2.61 (2.16, 3.12)	0.90 (0.73, 1.09)	1.20 (0.98, 1.45)	−3.27 (−5.20, −1.31)
20–24	4.54 (3.69, 5.47)	3.43 (2.79, 4.13)	1.78 (1.44, 2.12)	2.18 (1.76, 2.59)	−2.72 (−4.57, −0.83)
25–29	5.47 (4.65, 6.53)	4.96 (4.22, 5.93)	3.20 (2.62, 3.75)	2.89 (2.37, 3.39)	−2.76 (−4.61, −0.88)
30–34	7.82 (6.66, 9.34)	8.84 (7.53, 10.55)	7.31 (5.82, 8.59)	5.66 (4.51, 6.65)	−2.59 (−4.45, −0.70)
35–39	16.75 (14.26, 19.89)	18.31 (15.59, 21.74)	11.19 (9.03, 13.16)	11.09 (8.95, 13.04)	−2.70 (−4.59, −0.77)
40–44	26.77 (22.71, 31.77)	39.81 (33.77, 47.24)	21.70 (17.63, 25.84)	21.35 (17.35, 25.42)	−2.97 (−4.85, −1.06)
45–49	36.65 (31.1, 43.01)	70.86 (60.13, 83.15)	39.73 (32.41, 47.66)	32.73 (26.71, 39.27)	−3.28 (−5.16, −1.37)
50–54	70.02 (59.63, 81.20)	146.48 (124.75, 169.88)	76.23 (62.36, 91.74)	60.94 (49.84, 73.33)	−3.85 (−5.70, −1.96)
55–59	107.97 (92.14, 125.02)	248.42 (212, 287.66)	97.94 (80.24, 117.13)	103.27 (84.61, 123.5)	−3.77 (−5.60, −1.90)
60–64	141.16 (121.88, 163.80)	398.53 (344.10, 462.45)	146.60 (122.63, 172.89)	186.62 (156.11, 220.09)	−3.56 (−5.40, −1.70)
65–69	183.60 (161.28, 211.35)	670.86 (589.30, 772.27)	238.92 (201.75, 280.32)	339.46 (286.65, 398.28)	−3.42 (−5.27, −1.53)
70–74	228.07 (201.52, 269.60)	1209.52 (1068.72, 1429.76)	327.72 (278.88, 379.93)	684.81 (582.74, 793.90)	−3.36 (−5.23, −1.47)
75–79	227.78 (203.91, 266.13)	1996.22 (1787.07, 2332.29)	362.57 (312.72, 416.05)	1214.77 (1047.76, 1393.98)	−2.99 (−4.91, −1.03)
80–84	183.02 (162.01, 216.05)	3245.67 (2873.12, 3831.38)	417.37 (360.79, 470.96)	2188.93 (1892.2, 2469.96)	−2.62 (−4.56, −0.63)
85–89	94.90 (82.73, 113.52)	4948.81 (4314.41, 5919.86)	311.31 (269.92, 350.65)	3660.48 (3173.88, 4123.02)	−2.32 (−4.27, −0.33)
90–94	23.61 (19.99, 28.37)	6336.52 (5363.56, 7612.73)	99.59 (81.82, 114.88)	4438.08 (3646.14, 5119.37)	−2.61 (−4.56, −0.61)
95+	4.71 (3.88, 5.53)	7573.85 (6228.16, 8884.52)	24.43 (19.27, 28.39)	5469.91 (4316.36, 6358.26)	−2.36 (−4.33, −0.35)

EAPC, estimated annual percentage change; 95% UI, 95% uncertainty interval; 95% CI, 95% confidence interval;

a age-standardized mortality rate;

b crude mortality rate in each age group.

**Table 3:** Number of DALYs and DALY rate of ischemic and hemorrhage stroke in China in 1990 and 2019 and EAPC from 1990 to 2019.

**Characteristics**	**1990**		**2019**		**1990–2019**
	**DALYs [**×**10**^3^ **(95% UI)]**	**DALY rate [per 100, 000 (95% UI)]**	**DALYs [**×**10**^3^ **(95% UI)]**	**DALY rate [per 100, 000 (95% UI)]**	**EAPC in DALY rate [% (95% CI)]**
Overall^a^	33621.26 (29916.11, 38026.59)	4134.28 (3697.14, 4674.62)	45949.13 (39813.51, 52335.53)	2412.52 (2102.92, 2742.48)	−2.83 (−4.60, −1.02)
**Sex** ^a^					
Male	18106.11 (15236.54, 21193.12)	4656.60 (3971.22, 5403.45)	27103.24 (22271.00, 32864.28)	3052.05 (2535.20, 3643.00)	−2.34 (−4.17, −0.48)
Female	15515.14 (13247.44, 18060.26)	3707.97 (3178.76, 4330.48)	18845.89 (15670.19, 22244.53)	1876.80 (1566.50, 2204.53)	−3.38 (−5.09, −1.64)
**Age at diagnosis**^b^ **(year)**					
0–14	1000.60 (690.22, 1222.39)	309.87 (213.75, 378.56)	93.76 (74.16, 120.52)	41.71 (32.99, 53.62)	−2.81 (−4.63, −0.95)
15–19	289.79 (245.68, 338.60)	228.39 (193.63, 266.86)	93.73 (77.37, 110.31)	124.75 (102.98, 146.82)	−2.35 (−4.12, −0.55)
20–24	374.54 (313.19, 440.18)	282.89 (236.55, 332.46)	160.53 (130.96, 187.44)	196.08 (159.97, 228.95)	−2.35 (−4.10, −0.56)
25–29	416.25 (357.58, 484.11)	377.73 (324.49, 439.32)	271.18 (225.86, 313.11)	244.92 (203.99, 282.80)	−2.30 (−4.06, −0.50)
30–34	525.67 (456.13, 607.14)	593.90 (515.33, 685.94)	524.96 (430.75, 606.95)	406.64 (333.66, 470.15)	−2.46 (−4.27, −0.62)
35–39	979.04 (850.75, 1146.58)	1069.89 (929.70, 1252.97)	691.39 (568.48, 801.40)	685.26 (563.44, 794.29)	−2.74 (−4.57, −0.89)
40–44	1365.92 (1176.16, 1600.83)	2031.34 (1749.13, 2380.68)	1170.00 (968.13, 1360.51)	1151.05 (952.46, 1338.48)	−3.00 (−4.83, −1.13)
45–49	1662.60 (1422.57, 1934.50)	3214.62 (2750.53, 3740.35)	1929.92 (1601.52, 2278.78)	1590.16 (1319.57, 1877.61)	−3.52 (−5.34, −1.67)
50–54	2777.62 (2380.68, 3195.08)	5810.68 (4980.29, 6683.98)	3251.20 (2711.66, 3834.61)	2598.81 (2167.53, 3065.15)	−3.44 (−5.27, −1.59)
55–59	3755.79 (3257.84, 4306.20)	8641.45 (7495.75, 9907.84)	3706.89 (3103.92, 4384.77)	3908.59 (3272.81, 4623.36)	−3.26 (−5.09, −1.39)
60–64	4231.52 (3697.27, 4871.95)	11946.49 (10438.21, 13754.57)	4784.67 (4064.31, 5578.26)	6090.79 (5173.78, 7101.01)	−3.12 (−4.98, −1.24)
65–69	4606.71 (4074.41, 5244)	16832.80 (14887.77, 19161.43)	6532.17 (5620.85, 7479.65)	9280.79 (7986.01, 10626.96)	−3.12 (−4.99, −1.22)
70–74	4646.28 (4126.91, 5428.97)	24640.29 (21885.99, 28791.10)	7184.06 (6205.01, 8192.53)	15011.86 (12966.02, 17119.15)	−2.80 (−4.73, −0.83)
75–79	3660.84 (3305.67, 4225.99)	32083.10 (28970.44, 37035.99)	6241.81 (5440.97, 7098.04)	20913.03 (18229.85, 23781.80)	−2.49 (−4.44, −0.49)
80–84	2247.42 (1997.41, 2608.97)	39855.85 (35422.06, 46267.55)	5414.79 (4721.75, 6047.91)	28398.18 (24763.50, 31718.61)	−2.23 (−4.18, −0.23)
85–89	883.80 (774.71, 1045.89)	46089.93 (40400.56, 54542.74)	3014.92 (2630.56, 3360.95)	35450.59 (30931.16, 39519.39)	−2.50 (−4.45, −0.51)
90–94	170.58 (145.01, 203.47)	45771.85 (38910.70, 54598.58)	746.28 (623.15, 852.31)	33257.45 (27770.13, 37982.52)	−6.41 (−7.76, −5.04)
95+	26.27 (21.78, 30.70)	42215.02 (34991.08, 49324.92)	136.90 (108.76, 157.39)	30657.65 (24356.42, 35247.24)	−2.36 (−4.32, −0.35)

DALYs, disability-adjusted life-years; EAPC, estimated annual percentage change; 95% UI, 95% uncertainty interval; 95% CI, 95% confidence interval;

a age-standardized DALY rate;

b crude DALY rate in each age group.

**Figure 1:**
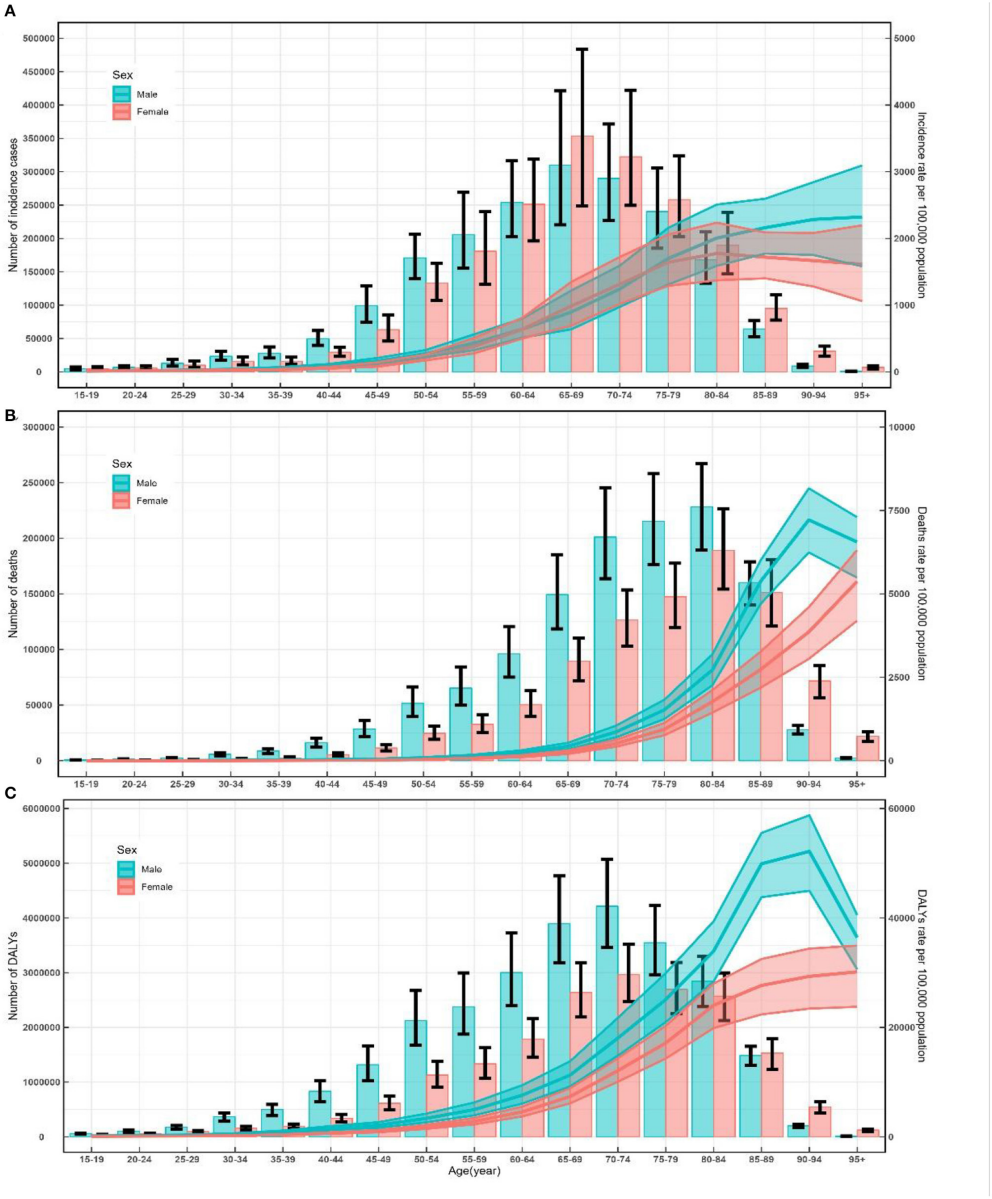
Number and rate of incidence **(A)**, death **(B)** and DALY **(C)** of ischemic and hemorrhage stroke by age and sex in 2019 in China. Shading represents the upper and lower limits of the 95% uncertainty intervals (95% UIs). DALYs, disability-adjusted life-years.

Among the total population in 2019, the numbers of incidence cases, deaths, and DALYs of ischemic and hemorrhage stroke reached a peak aged 65–69 years, 80–84 years, and 70–74 years, respectively (Tables 1–3), and these trends were similar for males and female patients (Figure 1). In contrast, the number of incidence cases, deaths, and DALYs was lower among men than women over 90 years old (Figure 1).

A peak in incidence, mortality, and DALYs was observed among the total population aged 80–84 years, 95+ years, and 85–89 years, respectively (Tables 1–3). The trends of age-specific rates of incidence among female patients were similar to the trends for the total population, whereas the trends of age-specific rates of incidence among male patients increased with increasing age. In male patients, the age-specific rates of mortality and DALYs peaked at 90–94 years old. In female patients, the age-specific rates of mortality and DALYs increased with increasing age. In addition, the numbers and rates of incidence, deaths, and DALYs were concentrated in the elderly population (≥60 years old) (Figure 1).

### Temporal trends of incidence, mortality, and DALYs of ischemic and hemorrhage stroke from 1990 to 2019

**Figure 2:**
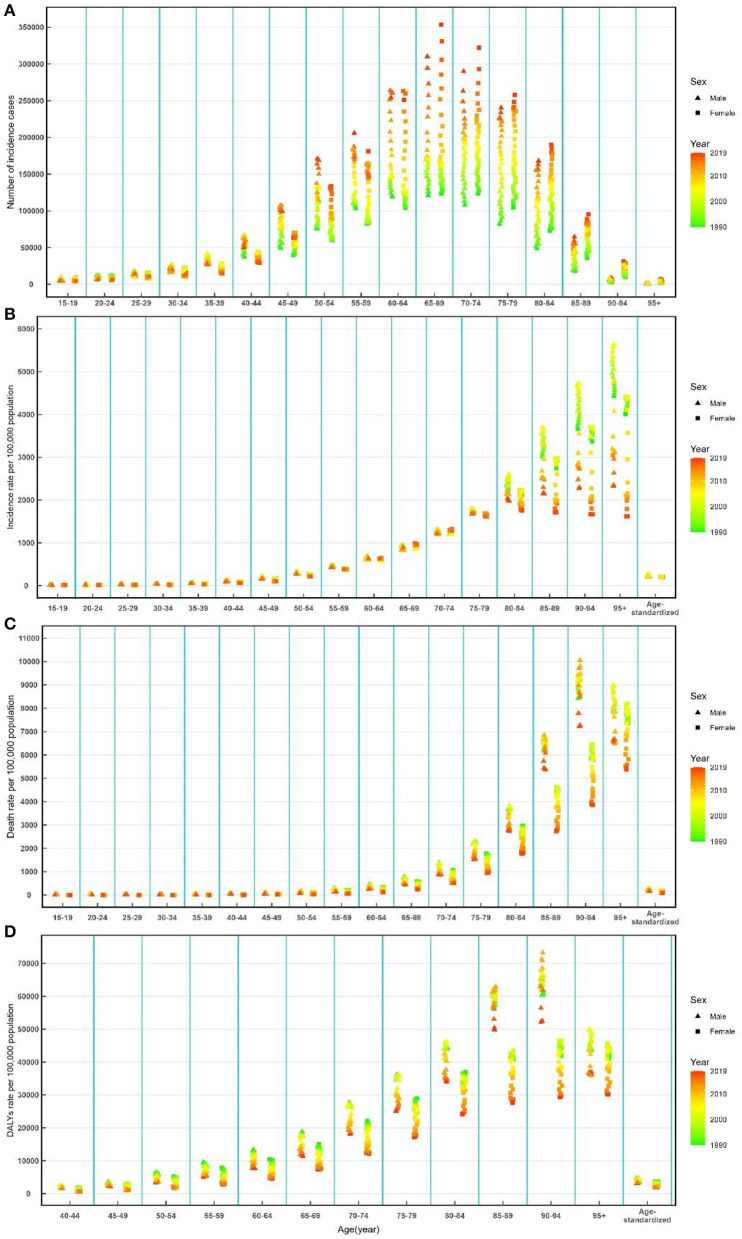
Number of incidence cases **(A)**, incidence rate **(B)**, death rate **(C)**, and DALY rate **(D)** of ischemic and hemorrhage stroke by age and sex from 1990 to 2019 in China. DALYs, disability-adjusted life-years.

There has been a significant increase in the number of incidence of cases, deaths, and DALYs of ischemic and hemorrhage stroke among the total population from 1990 to 2019 (Tables 1–3). The number of incidence cases increased by more than two times among men ≥60 years old and women ≥50 years old during the study period (Figure 2A). The ASIR was 221.51 per 100,000 (95% UI: 196.81, 249.61) in 1990, which decreased in 2019, with an EAPC of −1.31 (95% CI: −3.41, 0.85) in the total population (Table 1). The ASIR of female patients decreased more significantly than that of male patients during this period [EAPC = −1.43, 95% CI: (−3.51, 0.69) vs. EAPC = −1.20, 95% CI: (−3.35, 1.01), respectively] (Table 1). Additionally, overall downward trends in the incidence rates were observed among both sexes in most age-specific groups, while short-term upward trends were observed between 1990 and 2000 among male patients (Figure 2B).

The ASMR decreased from 1990 [211.44 per 100,000 (95% UI: 187.68, 243.80)] to 2019, with an EAPC of −2.85 (95% CI: −4.68, −0.98) (Table 2). A decreasing trend of age-standardized DALYs was also observed during this period, and the EAPC was −2.83 (95%CI: −4.60, −1.02) (Table 3). Overall downward trends in mortality and DALY rate were observed in most age-specific groups and both sexes from 1990 to 2019 (Figures 2C, D).

### Mortality and DALY rates of ischemic and hemorrhage stroke attributable to risk factors and their temporal trends from 1990 to 2019

**Figure 3:**
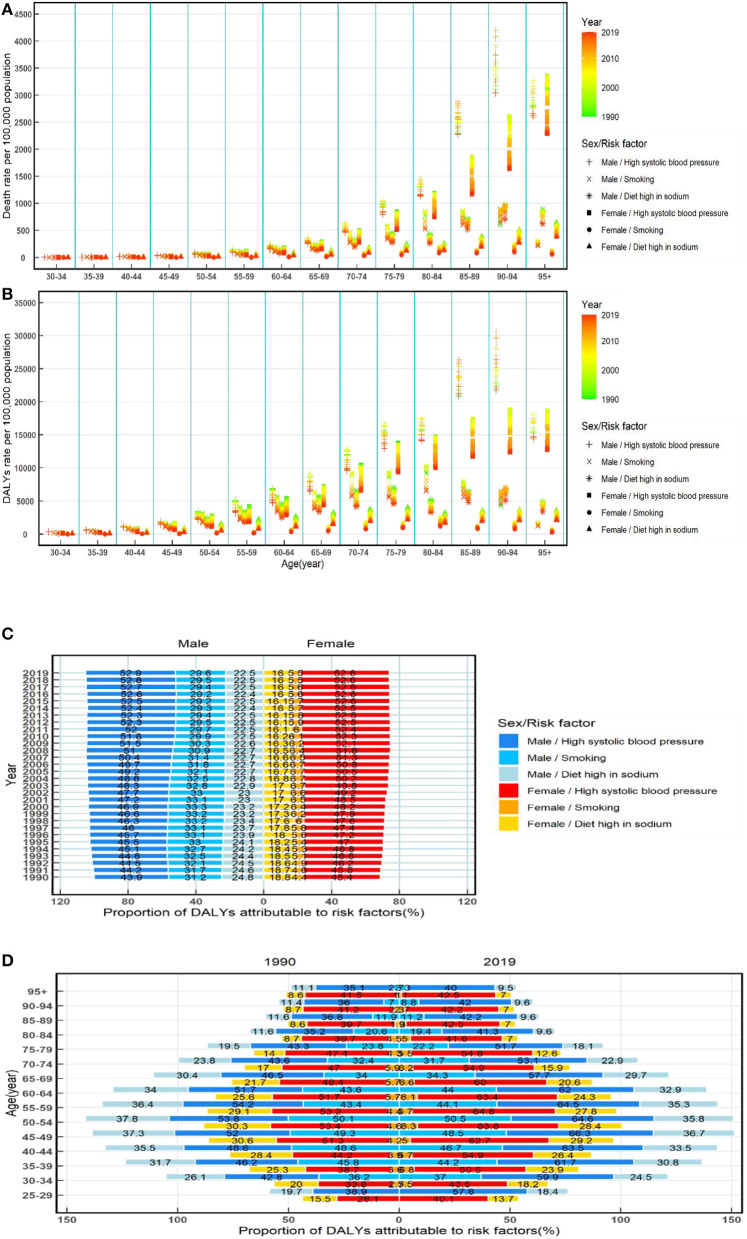
Rates of death and also rates and proportions of DALYs attributable to risk factors by age and sex from 1990 to 2019 in China. Rates of death **(A)** and DALYs **(B)** of ischemic and hemorrhage stroke attributable to risk factors by age and sex from 1990 to 2019 in China; proportions of DALYs attributable to risk factors by sex from 1990 to 2019 in China **(C)**; and proportions of DALYs attributable to risk factors by age and sex in 1990 and 2019 in China **(D)**. DALYs, disability-adjusted life-years.

The mortality that was attributed to high systolic blood pressure was the highest among men and women in all age-specific groups (Figure 3A). Trends of ischemic and hemorrhagic stroke mortality attributable to smoking were seen to grow at first and then decline, while decreasing trends of ischemic and hemorrhage stroke mortality attributable to smoking were observed in men in most age-specific groups. The overall downward trends attributable to high systolic blood pressure and a high-sodium diet were observed in most age-specific groups and both sexes (Figure 3A). Moreover, the temporal trends of the rates of DALYs attributable to smoking, high systolic blood pressure, and a high-sodium diet exposure were similar to those mortality (Figure 3B).

There was a difference between male and female patients in the proportion of DALYs attributable to risk factors (high systolic blood pressure, smoking, and high-sodium diet). High systolic blood pressure was the most significant contribution among both sexes, accounting for more than 43.9% of DALYs in male patients and more than 45.4% of DALYs in female patients, and the proportions of DALYs attributed to high systolic blood pressure increased over time from 1990 to 2019. For male patients, the proportion of DALYs attributable to ischemic and hemorrhage strokes was ~5 times higher than for female patients (Figure 3C).

Furthermore, the proportion of DALYs attributable to high systolic blood pressure, smoking, and a high-sodium diet shows a notable difference between sexes in most age-specific groups during this period, and there was a higher proportion of DALYs attributable to three risk factors among male than female patients. The proportion of DALYs attributable to high systolic blood pressure, smoking, and a high-sodium diet was observed having a relatively flat increase but then a drastic decrease in >65 years old among male and female patients (Figure 3D).

### Predictions of incidence and mortality of ischemic and hemorrhage stroke from 2020 to 2044

**Figure 4:**
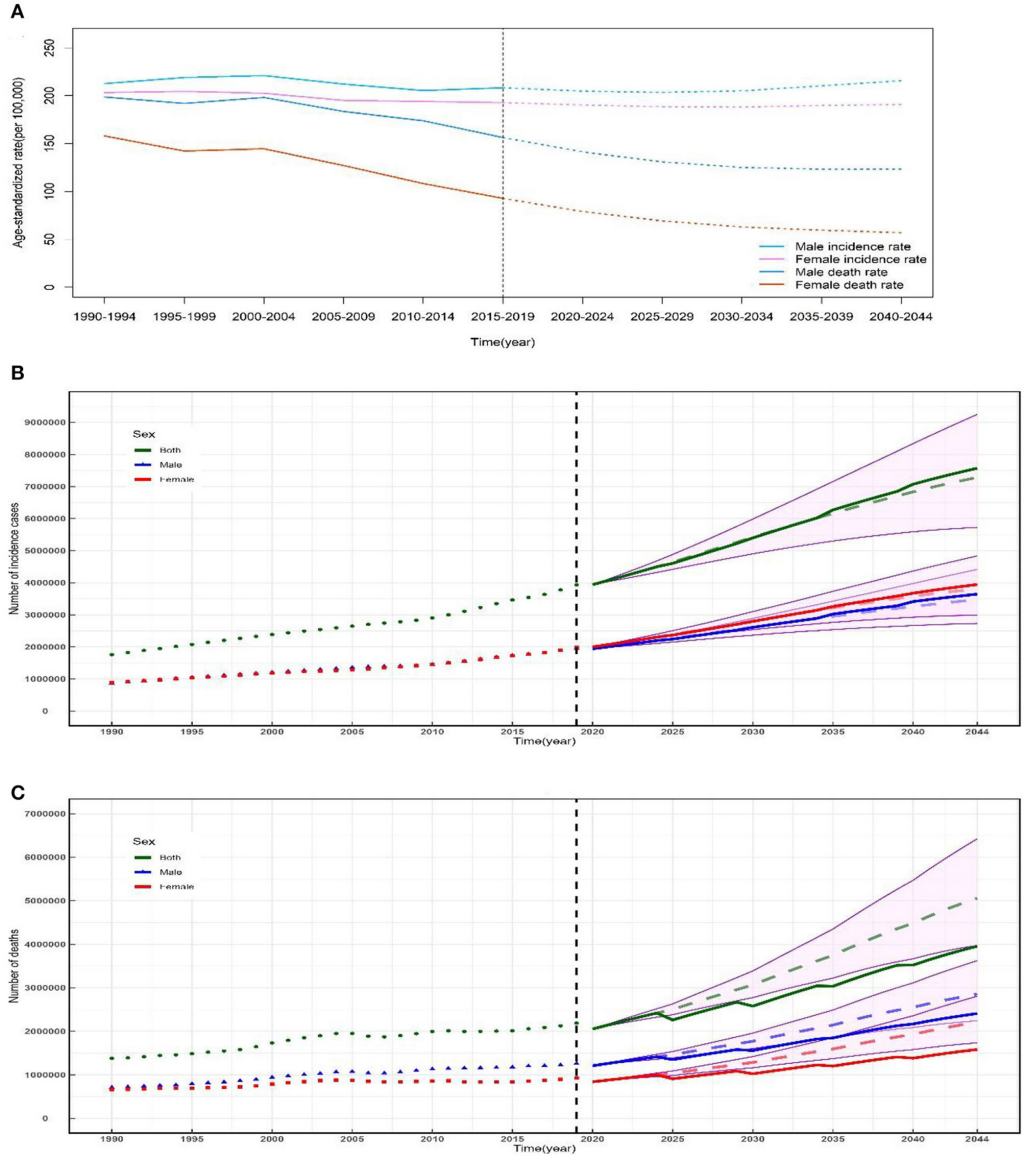
Temporal trends and forecasted rates of incidence and death **(A)** and number of incidence cases **(B)** and deaths **(C)** of ischemic and hemorrhage stroke by sex from 2020 to 2044 in China. Solid lines and dash lines represent the observed and the predicted number of incidence cases and deaths of ischemic and hemorrhage stroke; shading represents a 1% decrease and increase interval based on the 2019 rate. DALYs, disability-adjusted life-years.

Based on GBD data of ischemic and hemorrhage stroke from 1990 to 2019 in China, we further predicted the number and rate of incidence and mortality in the next 25 years (Figure 4). In the next 25 years, the rates of incidence among both sexes should show a stable trend (Figure 4A), and the mortality among both sexes should decline (Figure 4A), while the numbers of new cases and deaths of ischemic and hemorrhage stroke should rise steadily from 2020 to 2044 (Figures 4B, C). There should be 7570.95 thousand new ischemic and hemorrhage stroke cases (Figure 4B) and 3954.71 thousand deaths of NVHD (Figure 4C) in 2044. In 2044, among male patients, the number of incidence cases and deaths should increase to 3645.09 thousand and 2409.47 thousand, respectively (Figures 4B, C). Among female patients, the number of incidence cases and deaths should increase to 3946.26 thousand and 1582.75 thousand in 2044, respectively (Figures 4B, C).

## Discussion

The analysis of epidemiological trends and major risk factors of stroke as well as the prediction of future epidemiological trends in our study will be of great significance in reducing the incidence and mortality of stroke.

### Prevalence and prediction of disease burden of stroke in China

New epidemiological data of the Global Burden of Disease (GBD) 2019 study on stroke at macro- and meso-level geographic scales enable us to offer the most consistent, up-to-date, and comprehensive overview of the prevalence and risk factors for stroke nationally. First, our study has shown that this synthetical assessment of the numbers of incidence cases and DALYs lost from PAD demonstrates that there has been a remarkable increase among the total Chinese population over the past 30 years. The number of incidence cases will increase with aging and economic development, and China becomes one of the countries with the highest burden of stroke in the world. Advanced age, hypertension, smoking, and diet high in sodium were proved to be associated with a higher risk of stroke. With the global aging process continuing in the next several decades, the burden of stroke will probably increase substantially in the next several decades. This is consistent with the results predicted by our prediction model. For the next 25 years, it indicates that the number of new cases, deaths, and DALYs of stroke should keep on increasing among male and female patients. Meanwhile, all the incidence rates, mortality rates, and DALY rates showed an overall increasing trend. Despite the improvement of clinical professional level and current policies to reduce the burden of stroke morbidity, it is clear that the continued heavy burden of stroke in China may be shifting from chronic morbidity to mortality.

### Cause of increased prevalence of stroke disease

Our study indicates that the age-standardized prevalence and DALY rates of stroke are prevalent among the Chinese population based on the data from GBD 2019, which is similar to the level worldwide. Although the age-standardized prevalence and DALY rates decreased in China basically, the significant increase in stroke cases and DALYs in China draws more attention. The increased prevalence of stroke results from manifold reasons. First, recent studies have shown that the elderly population aged ≥60 years is estimated to increase to 300 million by the end of 2025. Such an increase in absolute numbers of stroke-related new cases, deaths, and DALYs from the data in our study can be explained by the ever-growing population of elderly people and longer life expectancies. Second, some projects were implemented by Chinese authorities for the populations with a high risk of stroke. In 2011, the National Stroke Screening and Prevention Project promoted stroke emergency interventions, including stroke screening, acute stroke units, emergency green channels, and early rehabilitation services. The number of basic hospitals offering emergency stroke interventions increased from 58 to 224 from 2010 to 2016 (13). These improvements have undoubtedly contributed to the declining case fatality rates and increasing newly detected cases that have been witnessed. Third, other primary drivers include medical techniques, while concurrent declining CVD mortality and improved cardiovascular care are also the major contributors. The popularization of image diagnosis technology such as CT and MRI and new technologies such as computed tomography perfusion imaging (CTPI) and diffusion-weighted imaging (DWI) may increase the incidence of stroke (14). Increased stroke prevalence can also be explained by notably different lifestyles compared with previous generations. The past 3 decades have seen an economic boom in China, with unhealthy lifestyles, such as smoking, high-fat diets, and sedentary lifestyles, being increasingly adopted. Consequently, metabolic risk factors among younger populations have also increased (15, 16). With the improvement of the changing public health awareness and lifestyles in China, there has been a decline in the rates of incidence, mortality, and DALYs.

### Risk factors of stroke disease

Many pathological and behavioral conditions have been shown to lead to a higher risk of experiencing a stroke. Targeting risk factors include, but are not limited to, hypertension, smoking, diet high in sodium, obesity, and lack of physical activity. Traditional risk factors remain highly prevalent in stroke survivors, among which high systolic blood pressure was the most common. High systolic blood pressure is the most important modifiable risk factor for stroke, with a direct, strong, continuous, and linear relationship between blood pressure and stroke risk. Overall, 23.2% (estimated 244.5 million) of the Chinese residents aged ≥18 years had hypertension and another 41.3% (estimated 435.3 million) had pre-hypertension according to the Chinese guidelines (17). A meta-analysis of 147 trials stated that blood pressure increase of 5 mm Hg diastolic or 10 mm Hg systolic was associated with a 40% increase in stroke risk (18). Even among those who are not defined as hypertensive, the higher the blood pressure, the higher the risk of stroke (19). These could explain the result of our study, and we found that the proportion of DALYs attributable to high systolic blood pressure was more than 52.9% in China in 2019 among both sexes. Since 2009, the Chinese government has incorporated hypertension management into community public service projects, and ~100 million hypertensive patients are under management; training covers 31 provinces, 3,90,000 primary medical institutions, and 1.84 million medical staff; quality control covers 15,000 institutions and 1.71 million patients; 26.66 million people completed the mission (17). The benefits of hypertension management in reducing the risk of stroke have also been supported in our results. Among both male and female patients, the proportions of DALYs attributable to high systolic blood pressure decreased in nearly all age groups from 2000 to 2019.

In addition to high systolic blood pressure, smoking and diet high in sodium were two significant contributions to stroke from 1990 to 2019. The precision of our estimation for risk factors of stroke prevalence in our study is consistent with former data, which is consistent with former data (20), implying the importance of proper control of these two risk factors. However, among Chinese adults, the current rate of smoking is as high as 28.3% (21), and giving up smoking is associated with a considerable reduction in risk of stroke, and the benefit seems to be apparent within 5 years (22–24). On the other hand, China belongs to a country with a high-salt diet (25). The “Report on Nutrition and Chronic Disease Status of Chinese Residents (2020)” shows that the average daily cooking salt of Chinese households in 2019 can reach 9.3 g, and the daily individual intake in the North of China is about twice of that in the South. Compared with lower salt intake, higher salt intake was associated with a 24% higher rate of stroke (26). In our study, the proportions of DALYs attributable to smoking and diet high in sodium were higher in male patients than in female patients, and smoking contributed to more than six times higher in the proportion of DALYs in male patients than female patients. The China Adult Tobacco Investigation Report (2015) shows that 52.1% of men and 2.7% of women smoke, contributing to the gender differences. For diet, the biological plausibility of the association between sodium chloride intake and stroke risk shows that an elevated sodium chloride intake induced a negative effect on endothelial function (27), oxidative stress (28), platelet aggregation (29), arterial stiffness (30, 31), left ventricular mass and function (32), and the development of vascular damage (33). The Chinese government announced the Implementation Rules for the Regulations on Hygiene Management in Public Places, which clearly stipulates that smoking is prohibited in indoor public places. Meanwhile, the authorities have also responded positively and put forward salt reduction targets in China's Medium- and Long-Term Plan for the Prevention and Treatment of Chronic Diseases (2017–2025) and the National Nutrition Plan (2017–2030). The benefits of smoking cessation and salt restriction have also been shown in our results. Among both male and female patients, all the ASIR, AMIR, and DALY rates decreased in the past 3 decades, and this trend will remain the same in the next 25 years.

### Actions for stroke disease prevention in China

Although the overall decreasing trends in the rates of incidence, deaths, and DALYs were observed in all age-specific groups and both sexes, the numbers of new cases, deaths, and DALYs lost showed an upward trend from 1990 to 2019. The total expenses of healthcare and treatment for CVD have increased rapidly since 2004, which is much faster than the increase in gross domestic product. Furthermore, a large number of individuals with stroke hospitalization have caused huge economic burden. A multifarious approach is recommended to alter this condition. The 2019 edition of China's guidelines for the prevention and treatment of hypertension proposes antihypertensive treatment to reduce the total risk of morbidity and mortality of stroke. In 2016, the State Council issued the protocol of “healthy China 2030” plan (34), which forcefully put forward “comprehensively promote the implementation of tobacco control.” In 2019, the authorities promulgated the implementation of healthy China action, requiring that the proportion of people protected by comprehensive smoke-free regulations should be no <30 and 80%, respectively, by 2022 and 2030, and smoking rate of people over 15 years old should be <24.5 and 20%. Additionally, healthy China 2030 put forward that it is urgent to guide a reasonable diet (34). Even so, greater efforts and special attention should be paid to targeted public health strategy making for stroke control.

### Limitations

Our investigation has several limitations to be announced. First, we only evaluated the disease burden of stroke at the national level but did not conduct some more details provincially. Additionally, one of these limitations was that the accurate assessment of stroke-related mortality could be challenged by the complexity of differentiating between deaths directly attributed to stroke and those resulting from its coexisting health conditions. Second, the inclusion of more data on risk factors for stroke would contribute to a deep insight into the epidemiology of stroke and a more scientific preventive policy. We could not evaluate the stroke burden caused by other important risk factors such as high fasting plasma glucose and obesity because of incomplete or missing corresponding data in the GBD database. Third, it has been described previously how the inevitable limitations of the GBD methodology affect related studies (35, 36). The GBD study cannot capture the most recent changes in health status, because of the time lags in the reporting of health information by the authorities. In this study, although the data used to estimate the prevalence were corrected, fitted, and filled through multifarious models, non-determinacy still needs to be under consideration when interpreting our results.

## Conclusion

This study has demonstrated that stroke is continuing to be a major healthcare challenge in China in the past 3 decades, especially in the elderly. An even larger number of stroke cases are to be expected, while the ASIR, ASMR, and DALY rate should show a downward trend among both sexes. It may lead to high care and treatment costs in the next 25 years. Traditional risk factors remain highly prevalent in stroke survivors, among which high systolic blood pressure was the most common. Our study results are valuable in drawing attention to the control and treatment of stroke, and more preventative, therapeutic, and rehabilitative strategies for stroke are needed to reduce negative health outcomes.

## Data availability statement

Publicly available datasets were analyzed in this study. This data can be found at: Institute for Health Metrics and Evaluation (IHME), Global Health Data Exchange (GHDx), 2019 Global Burden of Disease (GBD) study, https://vizhub.healthdata.org/gbd-results/.

## Ethics statement

Ethical review and approval was not required for the study on human participants in accordance with the local legislation and institutional requirements. Written informed consent from the patients/participants or patients/participants' legal guardian/next of kin was not required to participate in this study in accordance with the national legislation and the institutional requirements.

## Author contributions

DL: Writing—original draft, Writing—review and editing. QG: Writing—original draft, Writing—review and editing. MH: Writing—original draft, Writing—review and editing. YH: Writing—original draft. YO: Writing—review and editing. MC: Writing—review and editing. XZ: Writing—review and editing. XL: Writing—review and editing, Methodology, Software, Writing—original draft.
